# Supplementary material for: Complexome profiling of the Chlamydomonas psb28 mutant reveals TEF5 as an early PSII assembly factor
Source: Plant Cell. 2025 Mar 17;37(6):koaf055. doi: 10.1093/plcell/koaf055 (PMC12164590; doi:10.1093/plcell/koaf055)
Supplement: koaf055_Supplementary_Data [file koaf055_supplementary_data.zip › psb28_interactive.zip!/plots/intensity/qualityPlots/peptides/_Cre09.g390604.t1.1.html]

### Cre09.g390604.t1.1

Trivial name:   
SeqCoverage [median]: 24.00  
Mass: 27.49 kDa  
  
 

| Sequence | MissedCleavage | Mass [Da] | wtIntensity | muIntensity | Score |
| --- | --- | --- | --- | --- | --- |
| AAQQTLR | 0 | 786.43 | 274.90 | 1387.00 | 131.04 |
| IEQNPEGGLTAQDMEIAVTK | 0 | 2143.05 | 0.00 | 946.72 | 70.41 |
| LALDLNTDR | 0 | 1029.55 | 3155.00 | 10484.39 | 128.69 |
| SLTLGISAHQK | 0 | 1153.65 | 0.00 | 431.86 | 42.84 |
| VRGEDFSSYLAK | 1 | 1370.68 | 375.29 | 2486.81 | 73.85 |
|  |  |  | 3805.2 | 15736.8 |  |
